# Androgen receptor expression in endometrial carcinoma and its correlation with clinicopathologic features

**DOI:** 10.1186/s13104-018-3403-9

**Published:** 2018-05-10

**Authors:** Atif Ali Hashmi, Zubaida Fida Hussain, Amna Qadri, Muhammad Irfan, Sahar Ramzan, Naveen Faridi, Amir Khan, Muhammad Muzzammil Edhi

**Affiliations:** 1 Liaquat National Hospital and Medical College, Karachi, Pakistan; 2 Shaheed Zulfiqar Ali Institute of Science and Technology, Karachi, Pakistan; 3 Kandahar University, Kandahar, 3802 Afghanistan; 4 Brown University, Providence, RI USA

**Keywords:** Androgen receptor, Endometrial cancer, Endometrioid carcinoma, Serous carcinoma, Carcinosarcoma

## Abstract

**Objectives:**

Recent evidence suggests a role of androgen receptor expression as a prognostic and therapeutic biomarker in endometrial carcinoma, therefore in the present study we aimed to evaluate the frequency of androgen expression in different subtypes of endometrial carcinoma and its association with clinic-pathologic features.

**Results:**

18/89 (20.2%) cases of endometrial carcinoma showed positive androgen receptor expression. On the other hand, low, moderate and high androgen receptor expression was noted in 7/89 (7.9%), 10/89 (11.2%) and 1/89 (1.1%) cases respectively. 15/77 (19.48%) of endometrioid cancers and 3/7 (42.28%) cases of serous carcinoma showed androgen receptor expression; while none of the cases of clear cell or carcinosarcoma revealed androgen receptor expression. No significant association of androgen receptor expression with various clinicopathologic features of endometrial carcinoma was noted. We found that a significant subset of endometrial cancers express androgen receptor especially a serous cancers; therefore we suggest that androgen receptor expression testing should be done in endometrial carcinoma.

## Introduction

Endometrial carcinoma (EC) is one of the most common gynecological malignancies [1]. There are various histologic subtypes of EC including endometrioid, serous, clear cell, mucinous carcinoma and carcinosarcoma. These have been historically categorized into two major groups (type I and type II cancers) [2]. Endometrioid cancers are typically hormone (estrogen) driven and they are strongly linked to estrogen exposure [3]. On the other hand, recent evidence suggest that serous cancers may also be associated with hormone exposure [4, 5]. Progesterone and related compounds halts estrogen driven proliferation and they can be used in the therapy for low grade endometrioid cancers [6]. Androgens also display anti-proliferative effect in normal endometrium and therefore can theoretically play a role similar to progestins [7]. Recent studies revealed that loss of androgen receptor (AR) expression was found to be with poor survival in EC [8]. On the other hand, AR expression may serve as a potential therapeutic target in EC. Therefore, in the present study we aimed to evaluate the expression of AR in EC in our population and its association with various clinic-pathologic parameters.

## Main text

### Case selection

Total 103 cases of endometrial carcinoma were selected from records of pathology department archives. All patients underwent surgeries at Liaquat National hospital, Karachi from January 2012 till December 2017 over a period of 6 years. The study was approved by research and ethical review committee of Liaquat National Hospital and informed written consent was taken from all patients at the time of surgery. Hematoxylin and eosin stained slides and paraffin blocks were retrieved and new sections were cut where necessary. Slides of all cases were reviewed by two senior histopathologists and pathologic characteristics like histologic type, tumor grade, T-stage, lymphovascular invasion were evaluated. Moreover, representative tissue blocks of 89 cases were available for AR immunohistochemistry.

### Immunohistochemistry

**Fig. 1:**
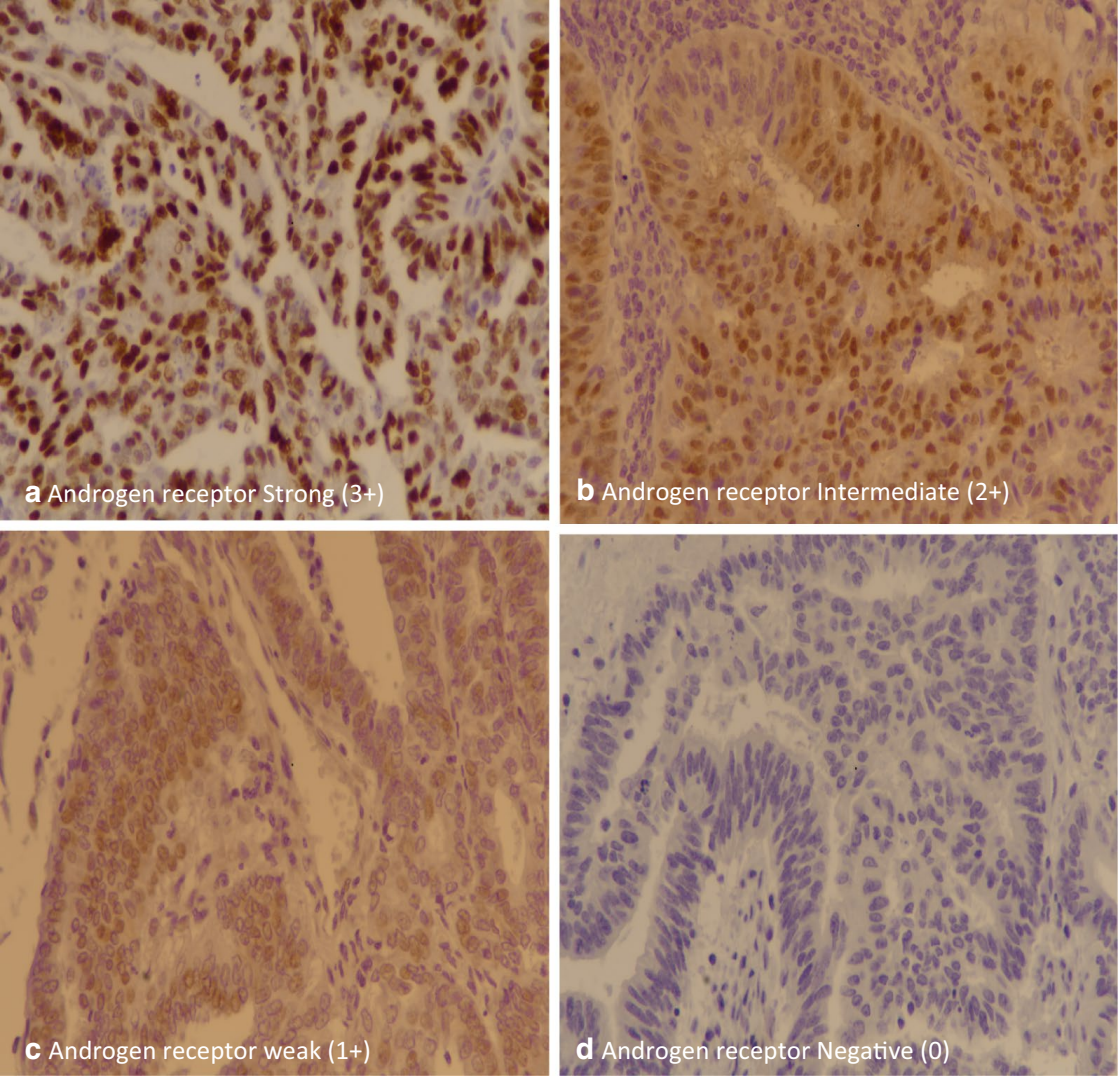
Androgen receptor expression in endometrial carcinoma

Androgen receptor IHC was performed using DAKO EnVision method using monoclonal mouse anti-human androgen receptor; clone AR441 according to manufacturer’s protocol (dilution of 1:50). Nuclear staining for AR was both quantitatively and qualitatively evaluated. Hormone receptor IHC scoring was based on the Liverpool endometrial steroid quick score with a final score out of 12 calculated by multiplying the proportion of positive tumor nuclei (1–10% = 1, 11–20% = 2, 21–40% = 3, > 40% = 4) by the staining intensity (0 = no staining, 1 = weak, 2 = moderate, 3 = strong). Scores of 1–4 were characterized as low, scores of 5–8 were considered moderate, and scores of 9–12 were classified as high (Fig. 1).

### Statistical analysis

Statistical package for social sciences (SPSS 21) was used for data compilation and analysis. Mean and standard deviation were calculated for quantitative variables. Frequency and percentage were calculated for qualitative variables. Fisher exact test was applied to determine association. P value ≤ 0.05 was taken as significant.

### Demographic patient’s profile

Table 1 shows demographic profile of patients. Mean age of the patients was 56.34 years (+ 9.79). 81.6% patients were post menopausal at the time of presentation. Most common histologic subtype of EC was endometrioid (87/103, 84.5%) followed by serous (9/103, 8.7%) and carcinosarcoma (6/103, 5.8%). Most patients had more than half of myometrial invasion (59/103, 57.3%), while cervical invasion, adnexal involvement and nodal metastasis was present in 26/103 (25.2%), 11/103 (10.7%) and 7/103 (6.8%) cases respectively. 89/103 (86.4%) cases were at stage T1/T2 while 71/103 (68.9%) were correspondingly at FIGO stage 1 (Table 1).

**Table 1:** Demographic profile of patients involved in the study (n = 103)

	n (%)
Age (years)	56.34 ± 9.79
Menopausal status	
Pre menopausal	19 (18.4)
Post menopausal	84 (81.6)
Histological type	
Endometrioid	87 (84.5)
Serous	9 (8.7)
Clear cell	1 (1)
Carcinosarcoma	6 (5.8)
Grade	
Grade I	41 (39.8)
Grade II	40 (38.8)
Grade III	22 (21.4)
Myometrial invasion	
Limited to endometrium	6 (5.8)
Less than half of myometrium	38 (36.9)
More than half of myometrium	59 (57.3)
Cervical invasion	
Present	26 (25.2)
Absent	77 (74.8)
Adnexal involvement	
Present	11 (10.7)
Absent	92 (89.3)
Nodal status	
N0	96 (93.2)
N1	6 (5.8)
N2	1 (1)
Lymphovascular invasion	
Present	8 (7.8)
Absent	95 (92.2)
T stage	
T1	72 (69.9)
T2	17 (16.5)
T3	10 (9.7)
T4	4 (3.9)
FIGO stage	
Stage IA	40 (38.8)
Stage IB	31 (30.1)
Stage II	18 (17.5)
Stage IIIA	8 (7.8)
Stage IIIB	2 (1.9)
Stage IV	4 (3.9)

### Androgen receptor expression in endometrial carcinoma

18/89 (20.2%) cases of EC showed positive AR expression. On the other hand, low, moderate and high AR expression was noted in 7/89 (7.9%), 10/89 (11.2%) and 1/89 (1.1%) cases respectively. 15/77 (19.48%) of endometrioid cancers and 3/7 (42.28%) cases of serous carcinoma showed AR expression; while none of the cases of clear cell or carcinosarcoma revealed AR expression. No significant association of AR expression with various clinicopathologic features of EC was noted (Table 2).

**Table 2:** Association of androgen receptor expression with clinicopathologic features of endometrial carcinoma

	n (%)					P value
	Negative (n = 71)	Low (n = 7)	Moderate (n = 10)	High (n = 1)	Total (n = 89)	
Menopausal status						
Pre menopausal	14 (19.7)	2 (28.6)	1 (10)	0 (0)	17 (19.1)	0.743
Post menopausal	57 (80.3)	5 (71.4)	9 (90)	1 (100)	72 (80.9)	
Histological type						
Endometrioid	62 (87.3)	5 (71.4)	10 (100)	0 (0)	77 (86.5)	0.117
Serous	4 (5.6)	2 (28.6)	0 (0)	1 (100)	7 (7.9)	
Clear cell	1 (1.4)	0 (0)	0 (0)	0 (0)	1 (1.1)	
Carcinosarcoma	4 (5.6)	0 (0)	0 (0)	0 (0)	4 (4.5)	
Grade						
Grade I	28 (39.4)	3 (42.9)	4 (40)	0 (0)	35 (39.3)	0.293
Grade II	30 (42.3)	2 (28.6)	6 (60)	0 (0)	38 (42.7)	
Grade III	13 (18.3)	2 (28.6)	0 (0)	1 (100)	16 (18)	
Myometrial invasion						
Limited to endometrium	4 (5.6)	0 (0)	2 (20)	0 (0)	6 (6.7)	0.518
< 1/2 of myometrium	26 (36.6)	3 (42.9)	2 (20)	0 (0)	31 (34.8)	
> 1/2 of myometrium	41 (57.7)	4 (57.1)	6 (60)	1 (100)	52 (58.4)	
Cervical invasion						
Present	17 (23.9)	3 (42.9)	3 (30)	0 (0)	23 (25.8)	0.581
Absent	54 (76.1)	4 (57.1)	7 (70)	1 (100)	66 (74.2)	
Nodal status						
N0	67 (94.4)	6 (85.7)	10 (100)	1 (100)	84 (94.4)	0.499
N1	3 (4.2)	1 (14.3)	0 (0)	0 (0)	4 (4.5)	
N2	1 (1.4)	0 (0)	0 (0)	0 (0)	1 (1.1)	
Lymphovascular invasion						
Present	5 (7)	1 (14.3)	0 (0)	0 (0)	6 (6.7)	0.529
Absent	66 (93)	6 (85.7)	10 (100)	1 (100)	83 (93.3)	
T stage						
T1	51 (71.8)	4 (57.1)	6 (60)	1 (100)	62 (69.7)	0.607
T2	10 (14.1)	3 (42.9)	3 (30)	0 (0)	16 (18)	
T3	7 (9.9)	0 (0)	1 (10)	0 (0)	8 (9)	
T4	3 (4.2)	0 (0)	0 (0)	0 (0)	3 (3.4)	
FIGO stage						
Stage IA	28 (39.4)	2 (28.6)	4 (40)	0 (0)	34 (38.2)	0.853
Stage IB	22 (31)	2 (28.6)	2 (20)	1 (100)	27 (30.2)	
Stage II	11 (15.5)	3 (42.9)	3 (30)	0 (0)	17 (19.1)	
Stage IIIA	6 (8.5)	0 (0)	1 (10)	0 (0)	7 (7.9)	
Stage IIIB	1 (1.4)	0 (0)	0 (0)	0 (0)	1 (1.1)	
Stage IV	3 (4.2)	0 (0)	0 (0)	0 (0)	3 (3.4)	

Fisher exact test applied

P value ≤ 0.05, considered as significant

In the present study, we aimed to evaluate the AR expression in endometrial cancers and found that a significant proportion of endometrial cancers especially serous cancers exhibit AR expression, that may have clinical and therapeutic significance.

Variable expression of AR was seen in previous studies. Sasaki et al. reported 21% expression of AR [9]; on the other hand, as high as 89% AR expression was detected in another study [10]. Some studies reported degree of differentiation/grade of EC to be inversely associated with AR expression [11]; however, no such association was noted in our study. In the prior studies most of the work focused on endometrioid EC, as it seems to arise as a result of hormonal drive. In a recent study, it was noted that 70% of serous cancers and 50% of carcinosarcoma also show AR expression and high levels of AR expression was noted in half of serous carcinoma [12]. These findings correlate with results of our study as we found 42.8% serous cancers to express AR, while high AR expression was also noted in serous carcinoma. Some studies also revealed association of AR expression with good prognostic features and better disease free survival [13]; however, we didn’t found any significant association of AR expression with various pathologic parameters like tumor stage and nodal metastasis.

From a clinical standpoint, it is important to know if AR IHC expression can identify a subset of EC that can benefit from anti-androgen therapy. Recent evidence supports this speculation that androgen receptor antagonism can be a therapeutic option in EC [14, 15]. This becomes especially important in high grade endometrioid and serous cancers in which endocrine (ER/PR) therapeutic option is not available. In our study we found a high frequency of serous cancers to express AR.

## Limitations

One of the major limitations of our study was lack of clinical follow up to elucidate AR expression with disease free survival and low number of cases of non-endometrioid cancer. Therefore, we suggest more large scale studies with clinical follow up to identify role of AR expression as prognostic marker in EC.

## Abbreviations

EC: endometrial carcinoma
AR: androgen receptor
